# Long-Term Effectiveness of Polymerized-Type I Collagen Intra-Articular Injections in Patients with Symptomatic Knee Osteoarthritis: Clinical and Radiographic Evaluation in a Cohort Study

**DOI:** 10.1155/2020/9398274

**Published:** 2020-07-22

**Authors:** Adrián Borja-Flores, Salvador I. Macías-Hernández, Gabriela Hernández-Molina, Andric Perez-Ortiz, Eloy Reyes-Martínez, José Belzazar-Castillo de la Torre, Laura Ávila-Jiménez, María Cristina Vázquez-Bello, Marco Antonio León-Mazón, Janette Furuzawa-Carballeda, Gonzalo Torres-Villalobos, Fernanda Romero-Hernández, Cidronio Albavera-Hernández, Jesús Pérez-Correa, Hilda A. Castro-Rocha

**Affiliations:** ^1^HGZ 5 Con MF, Delegación Estatal Morelos, Zacatepec, Morelos, Mexico; ^2^Orthopedic Rehabilitation Division, Instituto Nacional de Rehabilitación Luis Guillermo Ibarra, Mexico City, Mexico; ^3^Department of Immunology and Rheumatology, Instituto Nacional de Ciencias Médicas y Nutrición Salvador Zubirán, Mexico City, Mexico; ^4^Escuela de Medicina, Universidad Panamericana, Donatello 59, Insurgentes Mixcoac, Benito Juárez, Mexico City 03920, Mexico; ^5^Coordinación de Planeación y Enlace Institucional, Jefatura de Servicios de Prestaciones Médicas, Delegación Estatal Morelos, Cuernavaca, Morelos, Mexico; ^6^Centro de Investigación Educativa y Formación Docente, Coordinación de Planeación y Enlace Institucional, Jefatura de Servicios de Prestaciones Médicas, Delegación Estatal Morelos, Cuernavaca, Morelos, Mexico; ^7^Departments of Experimental Surgery and Surgery, Instituto Nacional de Ciencias Médicas y Nutrición Salvador Zubirán, Mexico City, Mexico; ^8^Department of Traumatology and Orthopedics, Hospital “Dr. Victorio de la Fuente Narváez”, Mexico City, Mexico; ^9^Siracusa No. 51 Col. Lomas Estrella, Mexico City, Mexico

## Abstract

**Objective:**

Polymerized-type I collagen (polymerized-collagen) is a downregulator of inflammation and a tissue regenerator. The aim was to evaluate the effect of intra-articular injections (IAIs) of polymerized-collagen among patients with symptomatic knee osteoarthritis (OA) in delaying or preventing joint replacement surgery. *Patients and Methods*. This was a cohort study of 309 patients with knee OA. Patients with mild-to-moderate disease were treated weekly with IAIs of 2 mL of polymerized-collagen for six weeks (*n* = 309). Follow-up was for 6–60 months. The primary endpoints included the following determinations: (1) therapeutic effect; (2) survival from total knee replacement surgery (TKR); (3) Western Ontario and McMaster University Osteoarthritis Index (WOMAC) and pain (visual analogue scale, VAS). Clinical improvement was defined as a decrease in pain exceeding 20 mm on the VAS and the achievement of at least 20% improvement from baseline with respect to the WOMAC score. Radiographic analysis was performed at baseline and 60 months. The joint space width in the medial, lateral, and patellofemoral compartments was calculated.

**Results:**

Patients who received IAIs of polymerized-collagen had a statistically significant improvement in the primary criteria (*p* < 0.05). Kaplan–Meier survival analysis of the therapeutic effect demonstrated 98.8% survival at 60 months with TKR as the endpoint. There was no significant reduction in joint space in any compartment based on the analyzed radiographs. No serious adverse events were recorded.

**Conclusion:**

Polymerized-collagen increased the time to TKR by at least 60 months, modifying the disease course, improving functional disability, and decreasing pain.

## 1. Introduction

Osteoarthritis (OA) is a disease entity that is characterized by alterations in the articular cartilage, subchondral bone, ligaments, capsule, and synovial membrane. In Latin America, according to the Community Oriented Program for the Control of Rheumatic Diseases (COPCORD), the prevalence of OA ranges from 2.3 to 20.4%, and the global burden of disease in individuals between 50 and 69 years is 679.55 years lived with disability per 100,000 habitants (http://www.healthdata.org) [1–4]. Nonsteroidal anti-inflammatory drugs (NSAIDs) and analgesics are the predominant modality of OA treatments, which at long-term or high-dose use may cause significant side effects, such as gastrointestinal bleeding, nephrotoxicity, and cardiovascular disease. However, intra-articular (IA) therapies employing diverse active drugs, such as corticosteroids and viscosupplementation, are also used [3, 4].

OA is considered an inflammatory disease [5–9], and interleukin (IL)-1*β*, tumor necrosis factor (TNF)-*α*, and IL-6 are the main proinflammatory cytokines involved in its pathogenesis. Moreover, IL-15, IL-18, IL-21, leukemia inhibitory factor 2 (LIF), chemokines (IL-8, MCP-1, RANTES), and adipokines have also been implicated [10]. Studies suggest that there is a window of opportunity early in OA's inflammatory process; thus, new therapeutic strategies to modify the structural progression of OA (disease-modifying OA drugs [DMOADs]) are needed. DMOADs in phase II/III/IV clinical development include oral salmon calcitonin, SD-6010, vitamin D3 (cholecalciferol), collagen hydrolysate, recombinant human fibroblast growth factor (FGF)-18, bone morphogenetic protein-7 (BMP-7), and avocado-soybean unsaponifiables (ASU) [11, 12].

For some years, our research group has focused on the effect of modified extracellular matrix proteins on the regulation of inflammation [13–23]. Particularly, polymerized-type I collagen is a *γ*-irradiated mixture of pepsinized porcine type I collagen and polyvinylpyrrolidone. The addition of 1% polymerized-type I collagen to cocultures of OA cartilage and synovial tissue induces cartilage regeneration due to a 3- to 6-fold increase in chondrocyte proliferation (Ki-67), proteoglycans, cartilage oligomeric matrix protein (COMP), and type II collagen [18]. Polymerized-type I collagen induces the downregulation of inflammation-inhibiting proinflammatory cytokine expression (IL-1*β* and TNF-*α*) through the modulation of NF-*k*B activation [15, 20]. In addition, polymerized-type I collagen downregulates the circulating number of Th22, Th17, and Th1 cells and upregulates IL-10 expression and the circulating number of regulatory T, B, and plasmacytoid dendritic cells [15, 18, 20], *unpublished observations*.

Previously, the effect on cartilage repair of four intra-articular injections (IAIs) of polymerized-type I collagen was evaluated in a rat model of early and long-term OA. The biodrug induced chondroprotection and high-quality cartilage repair (hyaline cartilage) and reduced degenerative damage in the cartilage structure [21]. In addition, the IA administration of 2 mL of polymerized-type I collagen to the knees of OA patients showed improvement in the Lequesne Index, Western Ontario and McMaster University Osteoarthritis Index (WOMAC) score, pain intensity according to the visual analogue scale (VAS), and patient global score at 6 months of follow-up, as well as 75% decreased daily consumption of NSAIDs. In addition, the patients who received this treatment had a threefold decrease in urinary CTX-II [19, 20]. Its clinical effect seems to be related to a decrease in proinflammatory cytokine- (IL-1*β*- and TNF-*α*-) expressing cells and an increase in IL-10-producing cells and CD4 T-regulatory cells [20].

The aims of the study were the following: (1) evaluating the long-term effectiveness and safety of polymerized-type I collagen IAIs in patients with knee OA; (2) determining the five-year therapeutic effect on knee survival, taking knee arthroplasty and arthroscopic knee surgery as endpoints; and (3) assessing whether polymerized-type I collagen improves the clinical and radiographic conditions of OA and delays and/or prevents joint replacement.

## 2. Patients and Methods

### 2.1. Study Design

This was an open-label, single-center study.

### 2.2. Patients

From March 2010 to June 2016, a total of 335 consecutive patients from Hospital General de Zona Número 5 con Unidad de Medicina Familiar, IMSS, received polymerized-type I collagen. All the patients were recruited from the IMSS National Program for deferral of total knee replacement surgery (institutional program to implement institutional strategies for nonsurgical treatment of knee OA).

### 2.3. Inclusion and Exclusion Criteria

All the patients fulfilled the American College of Rheumatology classification criteria for knee OA (knee pain with a duration of at least 3 months and at least five of the following: age >50 years; stiffness <30 min; crepitus; bony tenderness; bony enlargement; no palpable warmth; erythrocyte sedimentation rate (ESR) <40 mmh^−1^; RF <1 : 40; and synovial fluid signs of OA (e.g., clear, viscous, or white blood cell count <2000/mm^3^)) [22]. In addition, to be included, the patients had to have a body mass index (BMI) <40 kg/m^2^, a stable therapeutic regimen with NSAIDs, and a negative standard forearm skin test result for polymerized-collagen (evaluated 72 h after the administration of 0.2 mL of polymerized-collagen) [23].

**Figure 1:**
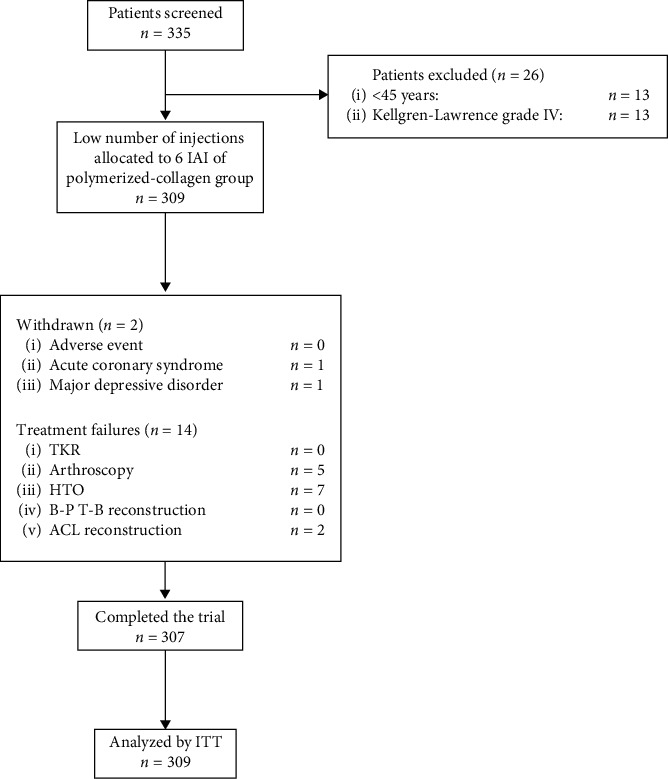
Enrolment and Outcomes. Course of the 60-month trial. ACL: anterior cruciate ligament reconstruction; B-P-T-B: bone-patellar tendon-bone reconstruction; IAI: intra-articular injections; ITT: intention to treat; HTO: high tibial osteotomy; TKR: total knee replacement.

We excluded patients undergoing treatment with oral, IA, or parenteral corticosteroids, as well as IA injections of any hyaluronic substance into the knee in the 3 months prior to the basal visit. In addition, we excluded patients with concurrent medical or arthritic conditions that could interfere with the evaluation of the knee joint, including fibromyalgia, Reiter's syndrome, rheumatoid arthritis, psoriatic arthritis, ankylosing spondylitis, lymphoma, arthritis associated with inflammatory bowel disease, sarcoidosis, amyloidosis, septic arthritis, crystal disease, Maquet high tibial osteotomy (HTO), bone–patellar tendon–bone (B–PT–B) reconstruction, anterior cruciate ligament (ACL) reconstruction, and cancer, as well as patients with disabling back or hip pain (Figure 1).

### 2.4. Intra-Articular Injections

IA injections were administered by either a lateral or medial approach after the instillation of 1 mL of 1% xylocaine solution at weeks 1, 2, 3, 4, 5, and 6 in the same knee. The most symptomatic knee was selected by the physician for treatment.

In case the patients had similar pain intensity in both knees, they were eligible to receive injections in both knees. The treating physician was board-certified orthopedic surgeon highly trained in IA injection techniques.

### 2.5. Clinical Evaluation and Efficacy Assessment

Patients' clinical records were carefully reviewed according to a preestablished protocol. The following data were collected for each study participant: demographic features and WOMAC score [24]. Pain intensity was evaluated on a 100 mm VAS (patient and physician). Each patient's and investigator's global assessment of disease activity on a 5-point rating scale, global assessment of disease activity change at the end of the treatment (Likert score), and assessment of therapy response were also determined. A clinical evaluation and efficacy assessment were performed at baseline and at 6, 12, 36, and 60 months during the study.

The primary endpoints were the WOMAC score, the pain on a VAS (patient and physician), and the need for total knee replacement (TKR) or arthroscopy of the knee due to OA. Secondary outcome measures included each patient's and investigator's global assessment of disease activity on a 5-point rating scale and global assessment of change in disease activity at the end of the treatment (Likert score: 0 = very poor; 1 = poor; 2 = fair; 3 = well; 4 = very well). The patient and physician assessments of therapy response were also determined (evaluation of medication: 0 = none: no good at all, ineffective drug; 1 = poor: some effect, but unsatisfactory; 2 = fair: reasonable effect, but could be better; 3 = good: satisfactory effect with occasional episodes of pain or stiffness; 4 = excellent: ideal response, virtually pain-free) [25]. Clinically significant improvement was defined as the decrease in pain exceeding 20 mm on the 0–100 mm VAS [22] and the achievement of at least 20% improvement from baseline with respect to the WOMAC score [24, 26–28].

### 2.6. Safety Assessment

Adverse events (AEs) were assessed at each visit. AEs were defined as any unwanted event occurring during the trial related to administration of the study biodrug. The assessment of each AE (local or systemic) was performed by the treating physician. At each visit, AEs were registered from a self-reported questionnaire as well as from clinical records.

### 2.7. Radiographic Analysis

Standard standing weight-bearing knee radiographic images were randomly obtained at baseline and 60 months after treatment. Measurement of the joint space in the anteroposterior and lateral compartments of the tibiofemoral and patellofemoral joints of the treated knee was performed. Radiographs were analyzed using a DICOM viewer (Poznan, Poland), which includes tools for radiological distance measurement. The radiological evaluation was carried out by two trained experts in musculoskeletal radiology. The intraclass correlation coefficients for the inter- and intraobserver evaluations were calculated (intra- and interobserver reliability were measured using the weighted kappa statistic and intraclass correlation coefficient [ICC]). *Kappa* <0.20 indicated slight agreement, 0.21–0.40 fair, 0.41–0.60 moderate, 0.61–0.80 substantial, and 0.81–1.0 almost perfect agreement [24]. The progression of OA was defined as a decrease in joint space width (JSW) of 0.48 to 0.50 mm [29]. The radiological staging was performed on the KL scale.

### 2.8. Treatment Failures

Patients treated with polymerized-type I collagen who received oral or parenteral corticosteroids or IA injection of steroids, glucosamine/chondroitin sulphate products, or any hyaluronic substance during the follow-up were considered as treatment failures. Patients who underwent TKR, arthroscopy, high tibial osteotomy (HTO), bone-patellar tendon-bone (B-PT-B) reconstruction, or anterior cruciate ligament (ACL) reconstruction were also considered as failures.

### 2.9. Ethical Considerations

The protocol was approved by the National Commission for Scientific Research (IMSS Ref No. 09-B5-61-2800/201700/1336). The study was conducted in accordance with the principles of good clinical practice and the revised Declaration of Helsinki, 1989. All the participants provided written informed consent.

### 2.10. Statistical Analysis

**Figure 2:**
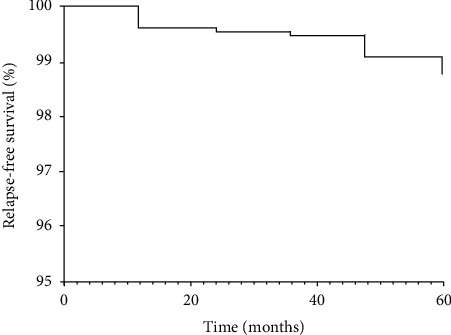
Covariate-adjusted relapse-free survival during the follow-up period (*n* = 309).

**Figure 3:**
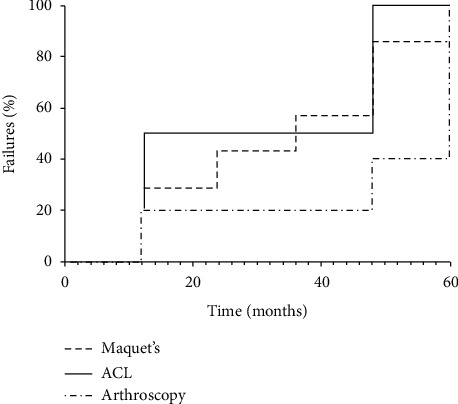
Failure function stratified by endpoint (*n* = 309).

We used descriptive statistics. For the primary analysis, the means of the scores between the two treatment groups were compared using an intention to treat (ITT) analysis. Multiple regression analysis was performed to assess associations between baseline characteristics and clinical outcomes. *p* values ≤0.05 were considered significant. Statistical analysis was performed using SPSS v.21 (SPSS Inc., Chicago, IL). For our survival approach, we estimated the unadjusted survival from TKR, arthroscopic surgery, or osteotomy by the Kaplan–Meier method and curves in SAS v.9.4 (Figure 2). We then performed Cox-regression modeling between baseline patient characteristics and outcome-free survival. For the clinical efficacy assessments (e.g., WOMAC, 100 mm VAS) at different time points, we implemented an extended Cox regression model with time-varying covariates. Finally, we reestimated the survival from endpoints using the Cox model (Figure 3). We assessed the proportionality of the hazards of all our models with the ASSESS statement in SAS.

## 3. Results

### 3.1. Patient Characteristics

At the beginning of the study, the national program for deferral of total knee replacement surgery included 335 patients. We excluded 26 patients as follows: 13 patients younger than 45 years and 13 patients with Kellgren-Lawrence (KL) grade IV.

Sixty-seven percent of all patients were women. The mean age at the time of intervention was 64.0 ± 8.7 years. Most of the patients had Kellgren-Lawrence (KL) grade II (*n* = 160, 52%), followed by KG III (*n* = 149, 48%). The demographic and clinical characteristics of the patients are presented in Table 1.

During the follow-up, 2 (0.6%) patients withdrew from the study (Figure 1).

### 3.2. Primary Clinical Outcomes

The WOMAC pain subscale score, WOMAC disability subscale score, WOMAC stiffness subscale score, patient pain on the VAS, and pain evaluated by a physician decreased significantly from baseline to 60 months (Table 1).

At 60 months, 295 (95.5%) patients who received the intervention responded to treatment, and none underwent TKR due to the persistence of symptoms. Five (1.6%) patients underwent arthroscopic surgery; 7 (2.3%) patients underwent HTO; and 2 (0.6%) patients underwent ACL reconstruction (Figure 1).

### 3.3. Secondary Clinical Outcomes

The measures of efficacy included each patient's and investigator's global assessment of disease activity on a 5-point rating scale (Likert Score) and their assessments of therapy response (Table 1). The Likert score improved considerably during the follow-up, reaching a significant difference (*p* < 0.05) between baseline and the posttreatment period (Table 1). The patients' and physicians' drug evaluations showed statistically significant differences after only 12 months following treatment (Table 1).

### 3.4. Radiological Evaluation

Radiographs of forty-one knees were analyzed, 21 of which were right knees. Patients were evaluated for KL grading before and 60 months after treatment. There was a significant difference between baseline and follow-up (*p* < 0.014) (Table 2). However, there was no significant decline in minimum joint space width in any compartment (Table 2).

### 3.5. Adverse Events

Throughout the five-year study period, there were no records of serious adverse reactions or long-term complications related to the IAIs of polymerized-type I collagen except for injection site pain lasting <24 h in all patients.

### 3.6. Concomitant Medication

Patients received stable doses of NSAIDs during the administration of polymerized-type I collagen. Thus, during first week of treatment phase, all patients received 1600 mg per day of ibuprofen. Then, during the follow-up, NSAIDs were taken as needed. Patients took on average 1-2 tablets per month of ibuprofen. None of the patients received corticosteroids, glucosamine/chondroitin sulphate products, or viscosupplementation during the study period.

### 3.7. Survival Analysis

To estimate the freedom from TKR, HTO, ACL reconstruction, and arthroscopy surgery of our cohort, we performed a thorough survival analysis with the latter as endpoints. At 60 months, the survival from a surgical procedure was 98.8% (Figure 2). Males had 2.4-fold hazard failing compared to females (HR: _1.25_ 2.38 _4.52_, *p* < 0.0001). Also, overweight, and obese in dividuals had a higher risk compared to normal weighted individuals (HR_OW_: _1.17_ 2.37 _4.72_, *p* < 0.0159; HR_OB_: _1.46_ 3.45 _8.19_, *p*=0.0047) (Table 3). At baseline, an increase in the WOMAC score by one unit significantly increased the risk of failure by 5% (HR: _1.00_ 1.05 _1.10_, *p*=0.0059). However, the changes over time of our symptom assessments, WOMAC and 100 mm VAS, were not predictors of failure. Arthroscopy had the most considerable freedom from outcome compared to HTO and ACL reconstruction (Figure 3).

## 4. Discussion

OA of the knee is a common cause of chronic joint pain. It affects 35% of individuals >65 years and is the leading cause of disability in this group of patients. Several therapeutic approaches have been used, including medication (oral, topical, intra-articular) and a core treatment program consisting of exercise, education, dietary advice, and biomechanical interventions, as well as surgery as the last resort [30].

Total knee replacement is an effective treatment for end-stage knee OA. However, this option is only suitable for a subset of patients with controlled comorbidities, patients who are older than 40 years, and patients who can afford the intervention [31]. Nevertheless, the number of total knee replacements is expected to increase in parallel with the ageing of the population, which highlights the associated future economic burden. For example, in our institution, the number of total knee replacements increased dramatically from 3,200 to 4,250 during the period from 2006 to 2017 [*unpublished data*].

Herein, we evaluated the clinical benefit of intra-articular polymerized-type I collagen and found that the weekly application of six injections decreased pain as well as the need for TKR and arthroscopy at the five-year follow-up.

There are at least two previous clinical trials that have evaluated the effect of collagen in OA treatment. One of them has investigated a medical device, MD-Knee (Guna S.p.a., Milan, Italy), containing collagen of porcine origin of molecular weight equal to 300,000 dalton, produced through a process of tangential filtration. It is a pure product, contaminant-free, with standardized chemical and physical characteristics. The purpose of an in situ introduction of collagen is to provide an effective bioscaffold. On the other hand, its degradation in the constituent amino acids constitutes a nutritional support for tissues of the other joint structures. MD-Knee preparation is equally effective in knee OA symptoms over six months after a five-week injection course as the HA formulation (SUPARTZ®) [32]. Moreover, 3 mL IAI of porcine type I atelocollagen (BioCollagen group) has been also demonstrated to alleviate knee pain in OA patients at 24 weeks [33].

Nonetheless, our patients showed clinical improvement according to the WOMAC and VAS scores at all the follow-up visits. Indeed, polymerized-type I collagen also had a clinical benefit in the group with severe knee OA, defined as patients with a WOMAC subscale of functional disability score ≥20 and EVA ≥50.

According to a meta-analysis of 74 randomized clinical trials of IA hyaluronic acid products vs. placebo in knee OA, saline solution had a placebo effect characterized by an approximately 30% reduction in pain that persisted for at least 3 months, while hyaluronic acid improved pain by 40–50% [34]. In the present study, the improvement in the VAS pain score ranged from 43 to 62% during the 60 months of follow-up. In addition, a previous meta-analysis of randomized controlled trials demonstrated that the administration of a single IAI of saline significantly improved short-term (≤3 months) and long-term (6–12 months) knee pain. Our long-term study diminished the possibility of the placebo effect [35]. We also found that the use of polymerized-type I collagen prevented the use of any other intra-articular treatment during the study period.

The other main outcome of our study was the need for TKR or arthroscopy. We described a 98.8% covariate-adjusted knee survival rate at 60 months (characterized by the absence of TKR or arthroscopy). In comparison, a study that evaluated the IAI effect of a high-molecular-weight hyaluronic preparation in knee OA reported a 67% knee survival rate (absence of TKR) at 60 months [36]. Our results suggest that polymerized-type I collagen is able to control the relief of symptoms and may act as disease-modifying OA drug that could slow or halt the radiographic evolution of the disease and prevent tissue damage [18–21]. Further RCTs with specific clinical or radiological endpoints are necessary to confirm these data. Thus, polymerized-type I collagen may be a therapeutic option for patients considered too young for arthroplasty, patients whose occupation precludes them from having arthroplasty, or patients who, due to health complications, cannot undergo surgery despite having an orthopedic indication.

Regarding the radiological results, significant progress was observed when they were evaluated for KL grading. Most of the patients advanced from stage II to III, but not from III to IV. Nonetheless, reduction in radiographic joint space width is recognized as a standard for demonstrating structural benefits in knee OA [37].

In the present study, polymerized-type I collagen prevented any significant change in joint space narrowing in the medial, lateral, and patellofemoral compartments at five years of follow-up. This suggests that the patients had no radiological progression, implying preservation of the hyaline cartilage.

In addition, it is important to highlight the low incidence of local and systemic AEs of the drug. This result is in agreement with the low incidence of AEs attributable to IA therapies reported in clinical trials in knee OA, with the main AEs being postinjection pain and swelling, as in our study [38]. Moreover, we previously reported that polymerized-type I collagen was a dominant cost-benefit alternative compared with NSAIDs in the treatment of symptomatic knee OA [39].

The main limitation of our study was our open design. It is well known that a placebo is effective in the treatment of OA, especially with respect to pain, stiffness, and self-reported function [35, 40–43]. Nevertheless, our study included a nonnegligible number of patients with symptomatic knee OA who had a better clinical improvement than that described for placebo in the literature, as well as a long follow-up period. Another limitation includes the low number of radiographs analyzed. However, a reduction in radiographic joint space width was determined indicating a structural benefit in knee OA and no progression of the disease.

## 5. Conclusion

Polymerized-type I collagen has an excellent long-term clinical outcome and a safe profile. The obtained data show that polymerized-type I collagen improves functional disability, decreases pain, and increases the time of surgical deferral of TKR, suggesting that it could slow or halt the radiographic evolution of the disease. It is a nonsurgical treatment option that certainly deserves future research.

## Figures and Tables

**Table 1:** Demographic and disease history at baseline and during follow-up of the study.

Demographics	Baseline	6–11 Mo	12–35 Mo	36–48 Mo	49–60 Mo
	(*n* = 309)	(*n* = 309)	(*n* = 255)	(*n* = 206)	(*n* = 48)
Gender					
Female; *n* (%)	208 (67)				
Male; *n* (%)	101 (33)				
Age (years); mean ± SD	64.0 ± 8.7				
(Range)	(45–82)				
Body mass index (kg/m^2^); mean ± SD;	23.9 ± 3.2				
(Range)	(18.2–34.6)				
Kellgren-Lawrence grade					
II; *n* (%)	160 (52)				
III; *n* (%)	149 (48)				
Deformity					
Valgus; *n* (%)	7 (2)				
Varus; *n* (%)	302 (98)				
*Clinical variables*					
WOMAC pain subscale; mean ± SD	6.1 ± 3.4	4.4 ± 3.1	3.4 ± 2.4	4.3 ± 2.2	3.6 ± 2.1
(Range)	(2–16)	(0–14)	(0–14)	(2–10)	(2–12)
Δ (%)		−**27.8**	−**44.2**	−**29.5**	−**41.0**
WOMAC stiffness subscale; mean ± SD	4.2 ± 2.0	3.2 ± 1.7	2.5 ± 1.5	2.8 ± 1.5	2.4 ± 1.1
(Range)	(2–10)	(1–10)	(0–12)	(2–8)	(0–6)
Δ (%)		−**23.8**	−**40.47**	−**33.3**	−**42.9**
WOMAC disability; mean ± SD	21.0 ± 4.9	19.1 ± 4.8	16.3 ± 4.9	17.2 ± 5.4	15.9 ± 5.5
(Range)	(2–36)	(10–32)	(2–30)	(6–32)	(4–32)
Δ (%)		−9.0	−**22.4**	−18.1	−**24.3**
Patient pain (mm); mean ± SD	56.2 ± 13.5	32.1 ± 15	35.5 ± 13.6	29.4 ± 11.7	20.4 ± 10.6
(Range)	(30–100)	(0–80)	(20–70)	(0–70)	(0–60)
Δ (%)		−**42.9**	−**36.8**	−**47.7**	−**63.7**
Physician pain (mm); mean ± SD	54.0 ± 12.6	29.7 ± 13.2	30.4 ± 13.9	25.1 ± 12.1	20.1 ± 10.6
(Range)	(30–100)	(0–80)	(20–70)	(0–70)	(0–60)
Δ (%)		−**45.0**	−**43.7**	−**53.5**	−**62.8**
Patient Likert score (cm); mean ± SD	1.8 ± 0.9	2.1 ± 1.0	2.3 ± 0.9	2.5 ± 0.8	2.9 ± 0.7
(Range)	(0–3)	(0–8)	(0–6)	(0–6)	(1–6)
Δ (%)		18.3	**27.8**	**38.9**	**61.1**
Physician Likert score (cm); mean ± SD	1.8 ± 1.0	2.2 ± 1.0	2.3 ± 0.9	2.6 ± 0.9	3.0 ± 0.7
(Range)	(0–6)	(0–6)	(0–6)	(0–6)	(1–6)
Δ (%)		**22.0**	**27.8**	**44.4**	**66.7**
Patient drug evaluation (cm); mean ± SD		2.4 ± 0.9	2.0 ± 0.9	2.2 ±0.7	3.1 ± 0.5
(Range)		(0–4)	(0–4)	(1–4)	(2–4)
Δ (%)			6.0	9.1	**29.2**
Physician drug evaluation (cm); mean ± SD		2.1 ± 1.0	1.9 ± 0.9	2.1 ± 0.9	2.8 ± 0.7
(Range)		(0–4)	(0–3)	(0–4)	(1–4)
Δ (%)			8.0	0.0	**33.3**

Bold numbers depict clinically significant improvement.

**Table 2:** Kellgren–Lawrence grading scale. The intraclass correlation coefficient for interobserver was 0.778 (*p* < 0.001) and the intraobserver 0.80 (*p* < 0.001).

		Baseline	Follow-up	Δ (%)	CI95%	*p*
KL, *n* (%)	II	20 (48.8)	9 (21.9)	−26.9	16.5–42	0.001
	III	16 (39.0)	23 (56.0)	17.0	5–27.2	0.05

**Table 3:** Extended Cox model of relapse-free survival during the follow-up period including time-varying covariates.

Characteristic	Adjusted HR (95% CI)	*p* value
Fixed effects		
Age (years)	**0.9190 (0.8850, 0.9530)**	**<0.0001**
Females (ref.) vs. males	**2.3790 (1.2510, 4.5220)**	**0.0008**
Baseline BMI (kg/m^2^)		
Underweight	0.0000 (0.0000, 0.0000)	0.9872
Normal weight	Ref.	–
Overweight	**2.3560 (1.1740, 4.7270)**	**0.0159**
Obese	**3.4610 (1.4620, 8.1900)**	**0.0047**
Arthrosis grade		
II	Ref.	
III	**2.6460 (1.3230, 5.2900)**	**0.0059**
Baseline clinical scores (U)		
WOMAC	**1.0500 (1.0010, 1.1020)**	**0.0442**
100 mm VAS-MD	0.8790 (0.5850, 1.3190)	0.5327
100 mm VAS-PT	1.1080 (0.7480, 1.6410)	0.6090
Time covarying effects		
WOMAC	0.9990 (1.9980, 1.0010)	0.5449
100 mm VAS-MD	—	—
100 mm VAS-PT	—	—

“—” means model did not converge. Significant predictors at the 0.05 level are displayed in bold, and those at the 0.10 level (marginally significant) are displayed in italics.

## Data Availability

The data used to support the findings of this study are available from the corresponding author upon request.
